# Lack of gastric emptying at autopsy eleven days after heat trauma in the sauna– a forensic autopsy case report

**DOI:** 10.1007/s12024-024-00931-3

**Published:** 2024-12-17

**Authors:** Susanne R. Kerscher, Natascha Kern, Nadezhda Chistiakova, Doreen Verhoff, Marcel A. Verhoff

**Affiliations:** Institute of Legal Medicine, University Hospital of Frankfurt, Goethe-University, Kennedyallee 104, 60596 Frankfurt/Main, Germany

**Keywords:** Hyperthermia, Full stomach, Gastrointestinal dysfunction, Burn, Post-mortem, Gastroparesis

## Abstract

A man in his mid-70s passed out in a public 90-degree sauna and remained unconscious for at least half an hour. He suffered third-degree burns to approximately 50% of his body surface area. Despite immediate transport to a burn center and intensive care therapy, he did not regain consciousness and died eleven days later.

When the body was opened, the lungs, liver, kidneys, and spleen showed changes consistent with the burns, intensive care therapy, and clinically suspected septic shock. The stomach contained approximately 200 ml of thickened chyme with coarse vegetable components. Such food components were not seen in the duodenum or in the following intestinal segments.

Considering the overall circumstances, the stomach contents must have been the last meal the man had eaten before the sauna session. The problem of reduced gastrointestinal motility in burn patients is clinically recognized. Nevertheless, a complete failure of gastric emptying for eleven days after intensive care therapy has not been described before and shows that the use of gastric contents in forensic practice is inappropriate for drawing conclusions about the time interval between last food intake and death and thus for estimating the time of death.

## Introduction

Gastrointestinal motility disorders have various causes, and the most clinically relevant manifestations are gastroparesis and functional dyspepsia [1]. Gastroparesis is classified as a disorder of gastric emptying in the absence of a mechanical obstruction in the downstream intestinal segments [2]. The two most common causes of gastroparesis are diabetes mellitus and idiopathic gastroparesis [3, 4]. More rarely, gastroparesis occurs postoperatively, is drug-induced or is caused by rare neuromuscular diseases such as neuropathies or Parkinson’s disease, metabolic, autoimmune, post-viral, rheumatologic, or paraneoplastic diseases [5]. Gastric emptying disorders have also been described in association with critical illness and intensive care therapy [6, 7]. Critically ill patients following traumatic brain injury and burns have been described to be at the highest risk of developing gastric emptying disorders [8–10]. The duration of physiologic gastric emptying has been reported to range from 45 to 90 min [11]. A study measuring gastric emptying time using the 13 C-octanoic acid breath test found a significantly longer half gastric emptying time (t_50_) of 155 min in critically ill intensive care patients compared to a healthy control group with t_50_ = 133 min [12]. Delayed gastric emptying of several days to a week has been described in forensic cases, particularly in association with severe trauma or physical shock [13–15].

Here we report on a man who, after eleven days of intensive care therapy and mechanical ventilation following severe burns, had an apparently full stomach with almost undigested food components at autopsy.

## Case report

A man in his mid-70s was found unconscious by other guests in a public 90-degree sauna. An infusion had taken place in this sauna at 15:00, in which the man had participated. He was still seen in the sauna at approximately 15:20. He was then found unconscious at approximately 15:50. The man was immediately rescued, and an emergency call was made. His upper skin had already peeled off during the rescue. The man was transported by helicopter to a burn center. He was diagnosed with third-degree burns over approximately 50% of his body surface area. He received intensive care therapy with catecholamines, massive transfusion, dialysis, prone ventilation, and repeated debridement. The man died eleven days after admission without regaining consciousness. His known medical history included dementia and hypertension.

A non-natural death was noted on the death certificate. A ‘septic shock’ due to a ‘wound infection’ due to ‘burns of approximately 50% of the body surface area’ is listed as a ‘disease leading directly to death’. Under ‘other major illnesses’, ‘acute renal failure’ and ‘ARDS’ (lung damage) are listed.

**Fig. 1:**
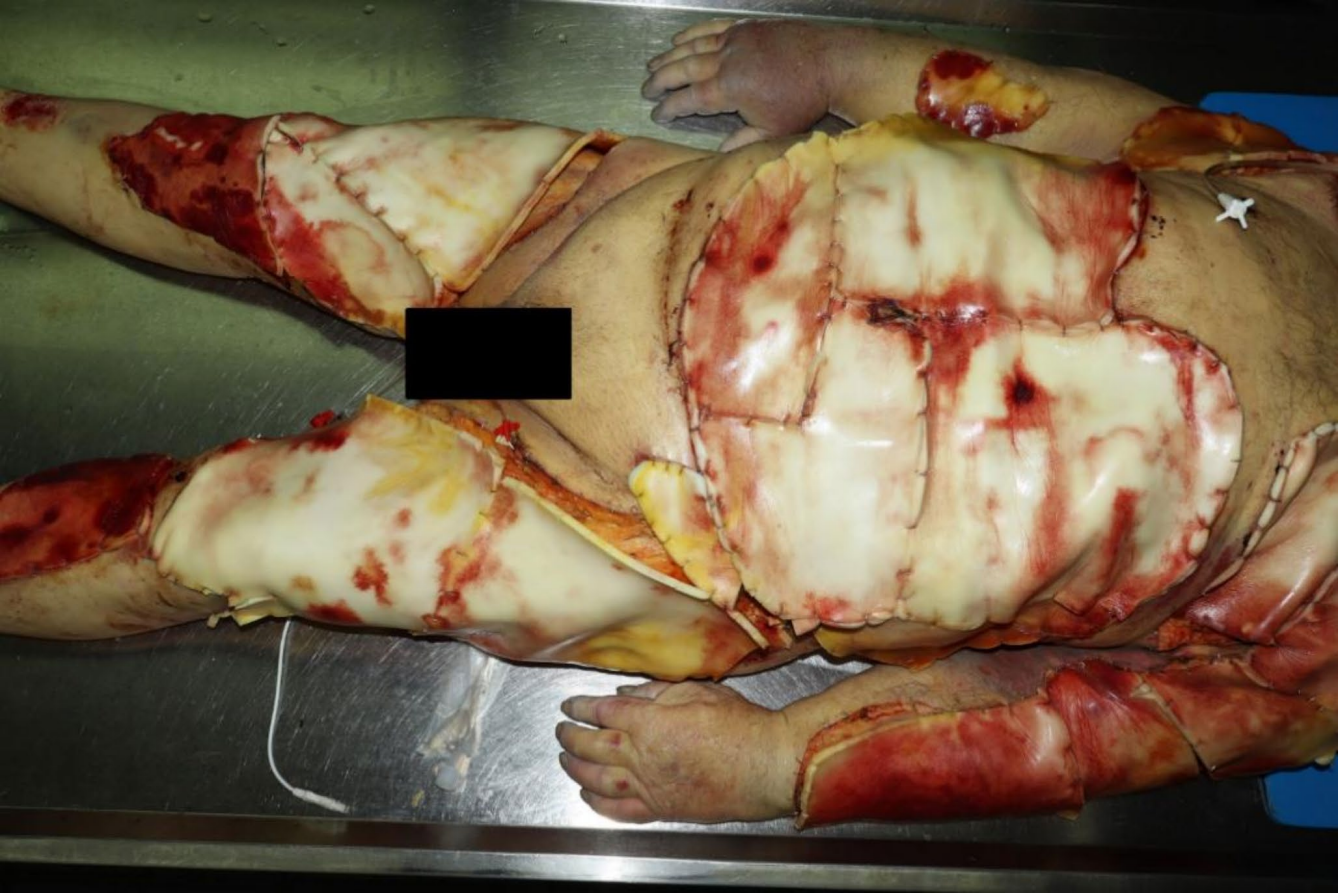
Severe burns (third-degree, 50% of body surface area) and surgical skin grafting eleven days after heat trauma in a public 90-degree sauna

### Autopsy results

The body of the elderly, overweight man was 161 cm long and weighed 98 kg, with a BMI (body mass index) of 37.8. The autopsy revealed extensive skin loss covered with artificial skin on the torso and the front of both arms and legs, as well as signs of burns on the skin of the face and the backs of both feet (Fig. 1). In addition, there were changes in the liver, kidneys, and lungs, indicating that the patient had been in an overload situation for some time following intensive care therapy and clinical suspicion of sepsis. In addition, there was a striking yellow discoloration (jaundice) of the body, both externally and internally. Acute cerebral edema had developed with pre-existing brain tissue atrophy and effusions into the abdominal and thoracic cavities.

**Fig. 2:**
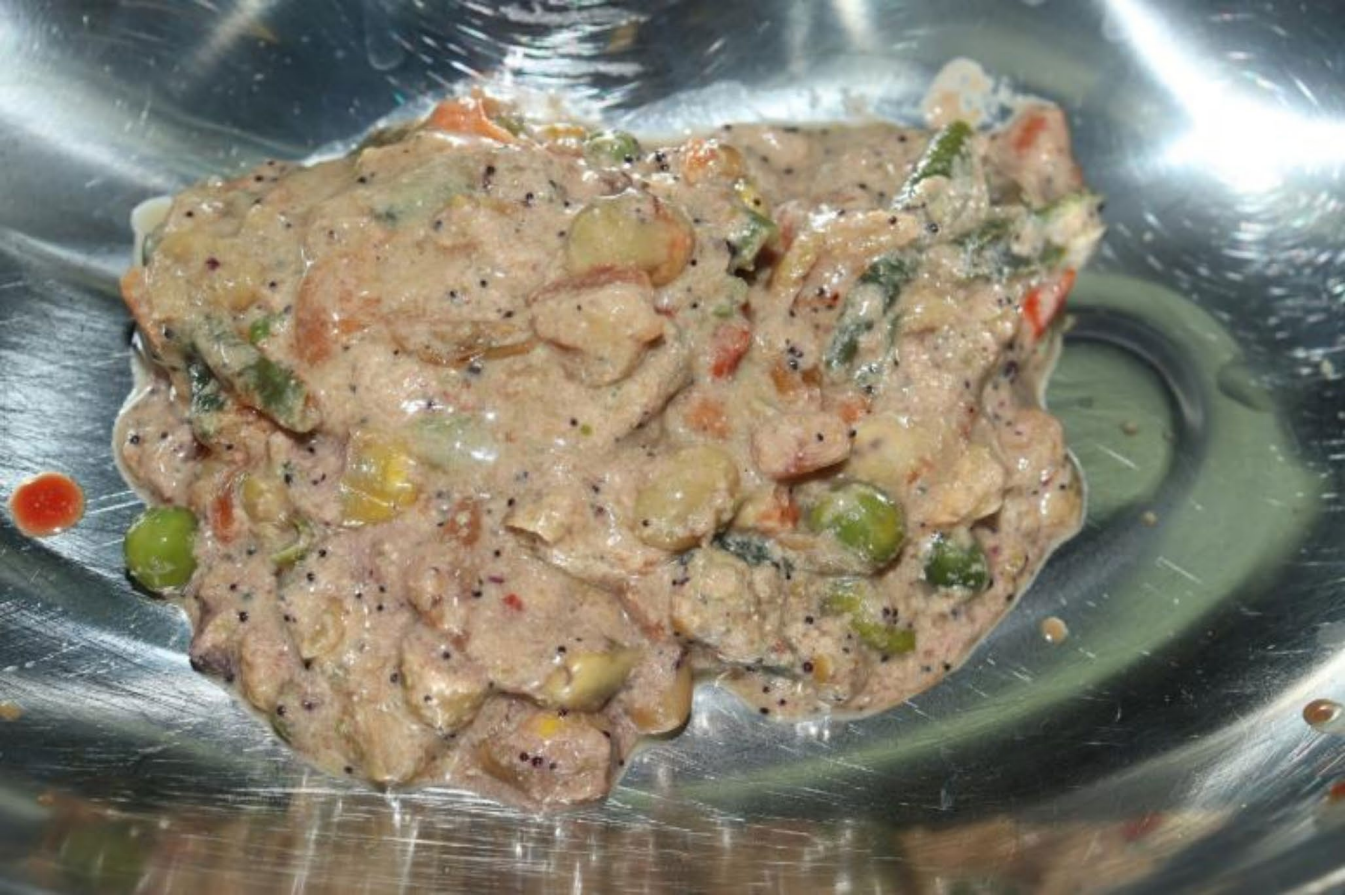
200 ml of gastric contents with solid, vegetable food components, eleven days after intensive care therapy with no intermediate consciousness

**Fig. 3:**
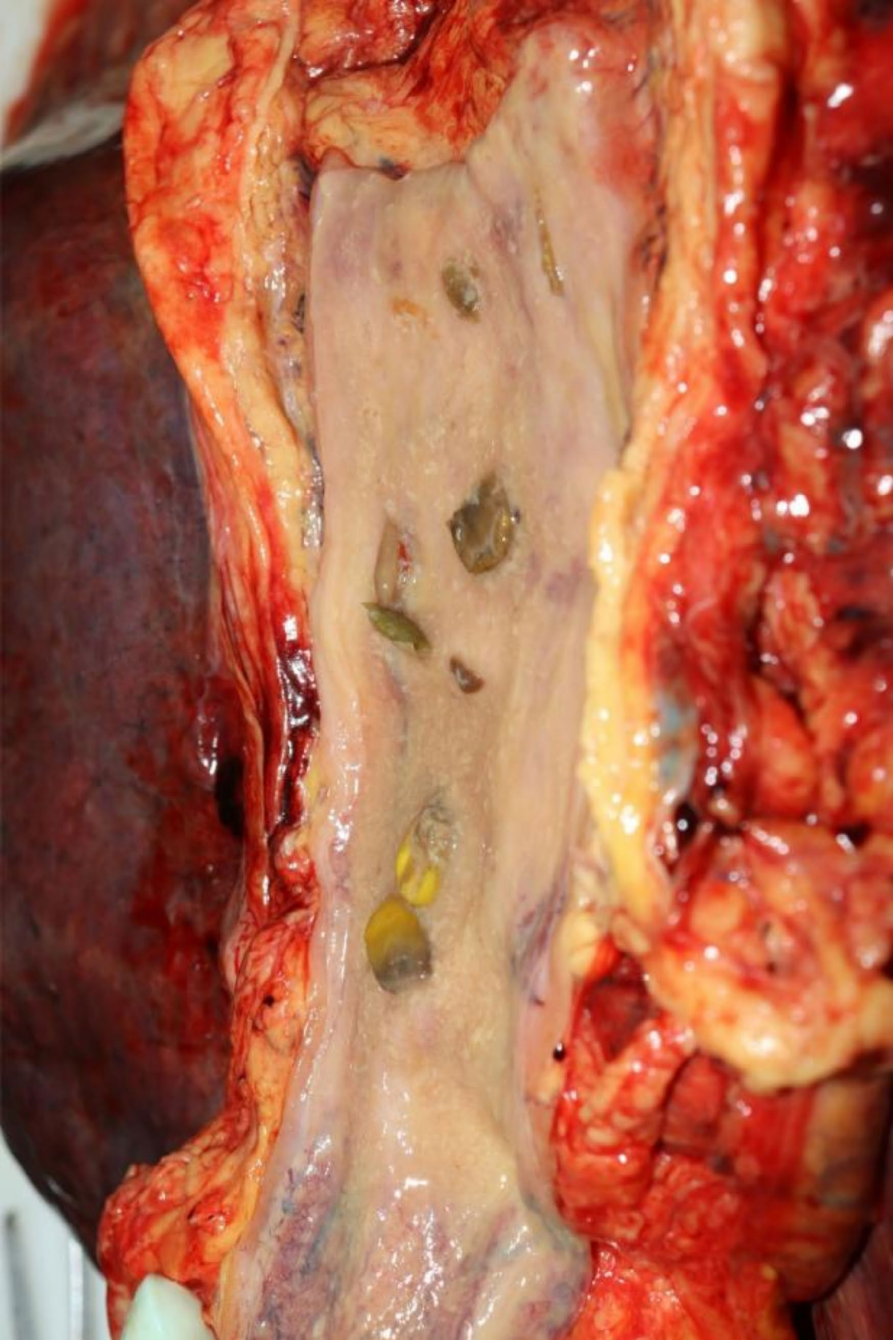
Single solid food components in the esophagus

A large amount (approximately 200 ml) of undigested gastric contents with coarse food particles reminiscent of vegetables could be seen in the stomach (Fig. 2). Solid food particles were also visible in the esophagus (Fig. 3). No such undigested food components were found in the duodenum or downstream intestinal tract. In addition, no mechanical obstruction was found in the downstream intestinal segments.

According to the macroscopic autopsy results, the cause of death was multi-organ failure. Based on the case history, this multi-organ failure was due to the severe burn injuries. The pattern of injury, with burns only to the front of the body, would be consistent with a prolonged period spent in a sauna in a seated position. It was a non-natural manner of death. Death was an indirect result of the effects of heat. The autopsy could not determine the cause of the unconsciousness in the sauna and showed no signs of external violence of fatal significance.

### Histology results

The results of the histopathological examinations were consistent with the macroscopic autopsy findings. The brain tissues showed edematous changes and signs of hypoxic brain injury, such as retracted Purkinje cell processes in the cerebellum and hypoxically altered cells in the cerebral cortex, basal ganglia and brainstem. There was no evidence of inflammation or new infarction in the heart tissue. Lung tissue showed mild signs of siderosis and fibrosis, but no pleural inflammation or signs of aspiration. Spleen, pancreas, kidneys and adrenal glands showed signs of autolysis. In the liver, cell decay and bile pigments in hepatocytes were observed, indicating intrahepatic cholestasis.

## Discussion

This article describes the case of a man who died after eleven days of intensive care therapy under mechanical ventilation after losing consciousness in a public sauna for unknown reasons and subsequently being exposed to extreme heat and suffering severe burns on his body. During the forensic autopsy, large amounts of almost undigested food residue were found in the stomach without any mechanical obstruction in the downstream intestinal segments. Because of the large amount of undigested food in the stomach, it could be mistakenly assumed that the man had eaten this food shortly before his death. However, since he did not regain consciousness between the sauna incident and his death, the stomach contents must have been his last meal eleven days earlier. Therefore, it can be assumed that gastric emptying was severely impaired or absent. The results of the histological examination were consistent with the autopsy findings and showed signs of multiple organ failure.

The main causes of gastroparesis are diabetes mellitus type 1 and 2 and idiopathic gastroparesis [1, 3, 4]. Metabolic, autoimmune, neuromuscular, rheumatic, or paraneoplastic diseases can also cause gastroparesis [5]. Gastroparesis is also known to occur postoperatively and due to medications (e.g. benzodiazepines and opioids) [16, 17]. The phenomenon of impaired gastric emptying has been described especially in critically ill patients in intensive care units [6, 7, 12, 18]. Ritz et al. conducted a study on 30 intensive care unit (ICU) patients and 20 healthy volunteers in which they measured the time to half gastric emptying (t_50_) using the 13 C-octanoic acid breath test. The time to half gastric emptying was significantly longer in critically ill patients than in healthy volunteers (t_50 =_ 155 min vs. 133 min), with the subgroup of burn patients showing significantly slower gastric emptying (t_50_ = 255 min) than the other groups [12]. Nguyen et al. performed a retrospective study of 132 ICU patients in which gastric emptying was assessed using the 13 C-octanoic acid breath test. Delayed gastric emptying was defined as t_50_ > 140 min. 60% of the patients had delayed gastric emptying with a mean t_50_ of 163 ± 7 min. Patients with burns were most commonly affected (77%) followed by patients with intracerebral injury (67%). This study also found an association between gastric emptying and older age, longer ICU stay and reduced renal function. Sex, ventilation pressure, type of sedation and BMI did not influence gastric emptying [7]. Another recent retrospective multicenter study evaluated 713 critically ill patients after heat stroke for gastrointestinal symptoms such as nausea and diarrhea. 18.5% of the patients had such symptoms. An impairment of the enteric nervous system due to heat exposure was suggested as a possible cause [19].

The main question in forensic pathology regarding gastric emptying is whether it can be used to determine the time between the last meal and death, and thus the time of death. In the forensic context, one factor that appears to influence gastric emptying primarily is the effect of physical stress during the digestive process [14]. Other factors associated with delayed gastric emptying in the forensic setting include increased intracranial pressure after traumatic brain injury, emotional stress, alcohol, and various drugs [20–22]. Delays in gastric emptying ranging from days to a week have been described in forensic cases [13–15]. Because digestion and gastric emptying are subject to many individual factors, it is generally not recommended to use them to estimate time of death [21, 23].

The development of gastroparesis appears to be a multifactorial process. In a study of 43 patients with symptoms compatible with gastroparesis, Abell et al. found evidence of increased inflammation with elevated serum levels of TNF-α and IL-6, electrophysiologic abnormalities in the sense of increased cutaneous electrogastrogram frequencies, and decreased Cajal cells [24]. Other studies have also described an association with impaired gastric motor function or an increase in inflammatory cytokines in critically ill patients as a possible cause of gastric emptying disorders [25, 26].

Regarding the duration of gastric emptying disorder, studies suggest that particularly severe trauma may delay gastric emptying for up to several days [13, 15]. One author describes the case of a traffic accident victim who was in a coma for one week after a fatal head injury. The subsequent autopsy revealed large amounts of undigested stomach contents [14]. Half gastric emptying times are generally reported to be in the range of 155–163 min in critically ill patients and 255 min in burn patients [7, 12]. A recent report published the case of a man who developed severe gastroparesis during inpatient treatment of osteomyelitis after surgery for diabetic foot syndrome. The duration of gastric emptying was 748 min [11].

The man in our case had only a history of hypertension and dementia and no other known pre-existing conditions that could lead to gastroparesis. The predisposing factors for gastroparesis in this case were severe burns on the body surface, eleven days of intensive care therapy with suspected administration of drugs, such as morphine derivatives, and clinical suspicion of developing sepsis and multi-organ failure. It can be assumed that the combination of these multifactorial aspects may have favored the development of a gastric emptying disorder. The reasons for the maximum extent of the gastroparesis over the long period of eleven days remain unclear. However, to our knowledge, such a long-lasting gastric emptying disorder has not yet been described in the literature before and is therefore unusual and rare.

In conclusion, this case provides further and recent evidence that the use of gastric contents in forensic autopsies is not reliable for drawing conclusions about the possible time between last food intake and death and thus for estimating time of death. Furthermore, this case provides a starting point for further, possibly post-mortem, studies to identify possible factors and modifiers of gastrointestinal dysfunction. In this way, knowledge of the pathophysiological correlates of intestinal function can be expanded.

## Key points

1. Gastrointestinal motility disorders have been described in critically ill intensive care and burn patients.
2. The pathophysiologic mechanisms underlying gastrointestinal motility disorders appear to be multifactorial and include immunologic, physiologic, and anatomic aspects.
3. Based on a physiological gastric emptying time of 45–90 min, the duration of gastric emptying dysfunction in previously described cases is up to one week.
4. We present the case of a man who died eleven days after severe heat trauma, and in whom the autopsy revealed the almost undigested last meal, in the sense of a severe, unusually prolonged gastroparesis.
5. This case demonstrates that stomach contents are inappropriate for drawing conclusions about the time between the last meal and death in a forensic context.
